# Indents

**Published:** 1915-08

**Authors:** 


### INDENTS

☞ **Brief Articles of Technical or Scientific Value will receive notice and comment. Contributions invited.**

*Supplying Missing Teeth.* It is my belief that a large per cent of the cases that come to us for this class of service call for a combination of splints, fixed bridgework and partial plates. One of the common mistakes that is made is the dependence upon either one or the other of these methods alone, rather than a combination of two or more. Rarely is it possible to meet the requirements in supplying missing teeth in mouths where there is a predisposition to pyorrhea with either fixed or removable appliances alone. Our plans should be so made that the minimum mutilation of the natural teeth will be required and yet when the conditions are such that the mutilation of several teeth is necessary to accomplish the fixation and support of one or more teeth, and to put the mouth generally in better condition, we should not hesitate to do so. We must consider the mouth and teeth as a unit and not individually. So that the question of conservation of the remaining natural teeth when partial artificial dentures of all sorts are being supplied is indeed a serious one.—F. E. Roach, in *Review*.

*Sterilizing the Tooth-brush.* I have recommended three manufacturers' products and made suggestions to patients as to how they may keep the tooth brush in a sanitary condition.

The suggestion is: take a glass tumbler, put brush in same with bristles downward and suspend the bristles by balancing the brush against the side of the tumbler and leave the handle pointing out. Put in a 50 per cent. solution of lysol. Let the brush remain in the solution one-half day. You will find sediment in the bottom of the tumbler. Remove the brush and shake out the solution from the bristles.

An ordinary pick used for teeth will serve, too, as an instrument, to pass through the bristles that run two ways and remove the sediment that has lodged on the back where the bristles are fastened. Now get a bowl of tepid water and shake the brush in same. Next saturate the bristles with full strength peroxide of hydrogen. You will notice a large amount of foaming. When the drug has exhausted wash out the bristles and repeat several times. The brush may now be put in the window where it can be exposed to sunlight and allowed to dry. A quart size fruit jar may be used as a sterilizer. Select a good, clean jar with a new rubber ring to make the jar air-tight. A small flat glass, one-ply, may be fitted to go into the jar at the largest part of the opening. This will not rest on the bottom but will be suspended. The length of the flat piece of glass can extend from the bottom to a point where the cap can be easily screwed on. A small board the width of which is no larger than the diameter of the jar, and the length of the jar, can be attached to the same. Tin strips can be used to bind the jar to the board to prevent movement of the jar when in use. Put a small dish under the flat piece of glass inside the jar containing a small piece of borax and a tablespoon of 37 per cent. liquor formaldehyde. Lay the brush on top of flat glass in the jar and screw on the top.

A one per cent. solution of formaldehyde will kill all pathogenic spores within an hour when exposed to the gas.

When the brush is removed from the jar containing the formalin gas, rinse with water before using as formaldehyde is intensely irritant to mucous membranes.—Dr. A. T. Landers in *Oral Hygiene.*

*Extracting Badly Broken Down Molars.* Take a cross cut fissure bur and separate the roots. In the upper molars cut mesiodistally, then bucco-lingually. In the lower molars cut bucco-lingually and extract the roots separately.

You will produce less trauma to the surrounding tissue, repair will take place in a shorter time, and it simplifies extraction to the minimum, especially when the roots diverge greatly or the roots are curved. If extraction is to take place under a general anesthetic I paint the field of operation with tincture of iodin, then proceed to separate the roots. When completed I then administer the anesthetic. If local anesthetic is used I first inject and by the time the roots are separated the parts are well anesthetized.—Fred F. Schwartz in *Review.*

*To Remove Stains from Necks of Teeth.* In cases where the gingival third of the tooth is coated with dark green stains, apply equal parts of tincture of aconite, tincture of iodin and chloroform. Then use a brush with pumice and, if necessary, repeat the application.—Dr. W. C. Smith, in *Oral Health.*

*Indirect Method of Inlay Making.* It has been thoroughly demonstrated beyond a doubt that no more accurate impression can be made with any material than with modeling compound if intelligently used, hence the modeling compound impression. The amalgam models are easily made, using the copper amalgam. Heat in ladle till mercury beads come out all over; put into mortar and work up as any other amalgam. Heat again if too dry, but do not burn it. If this does not bring out enough mercury add enough to make it plastic; then carefully pack it into your impression. This amalgam can be used over and over again. Crush the old models into small bits, and they can be reduced to a plastic state much better in the heating process.

Prepare your cavity as for an inlay in the direct method. Take a piece of casting wax and place into the cavity and have the patient bite into it; remove it and lay it away. This will serve as a guide after metal model has been made by slipping it into the cavity, giving you the occlusion and the contact point of the adjoining tooth. Now take a piece of thin metal and cut a matrix to come down to the gum and wide enough to come out past the margin bucco-lingually. The outer edges should be curved so as to force the material around these margins. Let the matrix extend above the occlusal surface sufficiently to clip it on either side. Bend a part of it back on the occlusal surface of the adjacent tooth; this will prevent the matrix from being forced into the gums. If necessary slip a wedge in at the cervix of the tooth. But do not let the matrix bind the margin of the cavity, for you want the soft compound to be forced out at all points, and the matrix to hold it against a portion of the tooth all around so that the margins will be clean cut. The matrix will prevent the compound from wedging around other teeth. With matrix in place take a piece of compound, in the form of a stick, and heat one end, leaving nard material behind it. Place in cavity, produce pressure so that it will be forced out over all margins. This soft material will follow the line of least resistance; make your cavity this line. Chill and remove.

Make metal model as directed, wax the bite to place by melting all around the margins, excepting the contact point and the occlusion. Cast and put it back in model; place in swager and hit it with hammer. Finish it off the model, trying it till satisfied, as polishing or grinding it on the amalgam model will bring out the mercury onto your gold.

My swager is a tube of steel with a plunger to fit and some soft rubber. See that your rubber is well about your model when swaging. If your technique is right your inlay will be perfect.—Miller W. Rice in *Review*.

*Silicate Fillings.* While I have not made the effort to determine how many silicious cement fillings I have made, my personal experience dates back to 1908; an experience which, though beset with some disappointments and some ignominious failures, has measured up enough results satisfactory to keep me at it. At no time have I discarded the silicious cement, and with its gradual improvement by the manufacturer, and the elimination of its earlier objectionable features, there has come more satisfactory and permanent results, and much pleasure in its use.

It is idle to hope that we may get away altogether from the use of gold fillings in the visible surfaces of the anterior teeth, we perhaps do not want to do so; and it is just as idle to expect to cease altogether the baking of porcelain inlays, yet we must all agree with the statement made by the essayist in the first paragraph of his paper, that today our patients demand that we disguise as much as possible the evidence of our operations. None of us likes the appearance of gold, and how many porcelain inlays, alas, have we seen whose margins in a few years develop an unsightly dark line following the outline of the inlay and cavity.

I am firmly of the belief that today, with our silicious cements in their present state of perfection, we have a filling material, which we may add to our list, and which has advantages that neither gold nor porcelain has, provided great care as to details be carried out in its manipulation.

It is my conviction that a dentist who can not put in a gold-foil filling which will pass muster, or who can not put in a measurably perfect porcelain inlay, will not get uniform results from the use of silicious cements.

The whole operation from start to finish is one of careful detail as to cavity preparation, dryness during filling, absolute cleanliness of slab, spatula and instruments, temperature of slab, selection of proper colors, mixing, and packing of filling and finishing of same. If any dentist will take up the silicious cement with a determination to use it conservatively and with painstaking care, and will make up his mind to read carefully and follow out the instructions given for his guidance by the manufacturer, I feel he will surely succeed with it and get results which will please alike his patients and himself.

Cavities in the gingival third of the incisors, cuspids, and bicuspids, and proximal cavities in the incisors and cuspids, not involving the incisal angles, are, as a general rule, the ones in which silicious cements are used to the best advantage. I would suggest to anyone having the fear that any sort of a contour or restoration, especially of the incisors, would be a dangerous procedure, that he try making some such restorations, first in molars and bicuspids in which the restorations can be bulky, and after gaining some assurance from watching these, to try the restoration of an angle in a bulky cuspid or central incisor.

In simple occlusal cavities in molars and bicuspids because of the four surrounding walls, there is no contra-indication for the use of silicious cements. Only a few days ago it was my pleasure to see seven such fillings, placed more than six years ago—all in fine condition—no discoloration—no marginal decay, and only the least appreciable bit of abrasion. In the simple occlusal cavities of the youngster's permanent teeth where we suspect later may follow proximal decays, we may safely use the silicious cements.

The gold inlay has its place, likewise the porcelain inlay, the gold-foil filling, the amalgams and the cements, but just as surely has the silicious cement come into its own, and to stay.—Dr. R. W. Parker in *Review*.

*The Unsanitary Tooth Brush and a Substitute.* To begin with I wish to endorse the paper by Dr. Feldman in the March *Oral Hygiene*. Now-a-days when the oral hygienists are urging the strenuous use of the tooth brush it seems to me a good time to condemn the use of that filth carrier *in toto*. That all tooth brushes are unsanitary must be admitted by all. At the risk of being charged with *a priori* reasoning I make the statement without fear of contradiction for the fact is obvious. No one, I think, would wash his face with a sponge or cloth so unspeakably nasty as is an ordinary toothbrush. How can an article of daily use in the mouth be anything but saturated with infection? If this be so how can the teeth be cleansed and disinfected by the use of a brush which is itself unclean?

Moreover the brush is a very inefficient agent anyway: Witness the many mouths of our patients who insist that they use the brush twice or thrice daily, and yet their teeth are far from clean. But the indictment against the brush is not merely negative. Far from it. Every dentist has seen cases where the roots of the teeth have been denuded of gum tissue—the gums have been literally scrubbed off the roots, exposing the roots to decay above the enamel margin. Enquiry will usually disclose the fact that the patient uses a very stiff brush, very vigorously. Once the attachment of the gum is broken pyorrhea steps in to finish the job. Now that recent discoveries have shown that pyorrhea is due to the presence of amœba, it is safe to say the brush is responsible for the spread of the disease by introduction of the amœba into lacerations of the gum margin.

To what extent the spread of pyorrhea is due to the brush it is impossible to say, but it is at least chargeable with a great deal. Is it too much to say, the more the brush is used the more pyorrhea there is to combat? I think not.

Of course, I would not condemn the brush unless I had a better thing to offer. I have. Take a piece of cheese cloth (surgeon's gauze) four inches square, wrap over the end of the forefinger, spread upon it the usual dentrifice and rub the surface of each tooth and the gums with it. After using once the cloth is thrown away. Followed by the floss between the teeth it is far more effectual than the brush, is cheap and cleanly and besides massages the gums thoroughly, and no possible harm can come from its use.—D. W. Barker, in *Oral Hygiene.*

The notion that a rag is a proper substitute for a tooth brush is rubbish. There is as yet no good substitute for the tooth brush. It can be kept clean enough for all practicable purposes, and when necessary it can be completely sterilized. We wonder if Mrs. Barker throws away her dish cloth after once using.—*Ed. Register.*

*Lack of Legal Dentists in England.* The alleged shortage in the number of qualified dentists was further discussed by the General Medical Council at their recently held Summer Session. It was decided that the Council's consideration of the matter should be deferred for the present, as only about half the replies had so far been received to their letter inviting opinions or recommendations from the teaching and examining bodies. The answers received had been summarized and presented in the report of the Dental Education and Examination Committee, but on the motion of Dr. Newsholme (whose resolution at the last Session had brought the subject before them) it was decided to delete from the report all reference to the nature of the replies that had so far come in. The expressed opinion of one-half of the bodies concerned might not correctly represent the views of the remaining bodies who had not yet replied, and the premature announcement and discussion of the opinions first received might "give a lead or an unwitting bias" to those authorities who were carefully considering the subject. In keeping with the spirit and intention implied in the Council's unanimous decision, we have omitted from our published report of the meeting the paragraphs which refer to the answers so far received.

It should be noted that Dr. Newsholme made a point of insisting that by the terms of his original motion the subject before the Committee was not the question of legislation, but whether, given that the law remained for the present as it now stood, it was practicable, without lowering the status and efficiency of the dental profession, to make any alteration in the curriculum. As thus stated, it might seem—as Dr. Newsholme wished to imply—that the Council had before it a clear-cut field of inquiry, and that the various teaching and examining bodies were invited to give or suggest solutions of a quite definite problem. But it will be remembered that according to the original motion, modification of the curriculum was to be considered solely with the view of *making good an alleged deficiency in the number of qualified dentists.* It is not likely then that the various bodies concerned will consent to be tied down to an expression of opinion limited in the way suggested, or that they will be silent as to alternative methods of augmenting the ranks of qualified practitioners. As to the regulation of dental practice, there are signs that certain representative authorities have come to believe that in this enfranchised country it may be expedient to interpret the "various bodies concerned" in a wider sense than has hitherto been accepted.—*British Dental Record.*

*Deaths from Anesthetics in England.* During 1913, the number of deaths under or connected with the use of anesthetics was 296, against 283 in the previous year. In 111 cases the kind of anesthetic is not stated, but in the remaining cases the anesthetics are specified as follows: Chloroform, 110; chloroform and ether, 25; ether, 25; A. C. E. mixture, 7; nitrous oxide, 7; ethyl chloride, 5; hedonal, 2; cocaine, 2; alcohol and chloroform, 2; stovaine, 1.

Of fatalities other than those under anesthetics, there are between seven and eight hundred annually due to poisonous substances. There were 190 fatal cases in which poisonous substances were taken accidentally, while in 470 cases the poisons were taken by suicides. Among the "accidental" cases the poison which accounted for the greatest number of deaths was laudanum, no fewer than forty-eight deaths being caused by this poison or by some other preparation or derivative of opium; to carbolic acid, taken in mistake for some harmless substance, ten deaths were due, and ten deaths to preparations of belladonna.—*Dental Record.*

